# Supplementary material for: Combined Therapy of Low-Dose Angiotensin Receptor–Neprilysin Inhibitor and Sodium–Glucose Cotransporter-2 Inhibitor Prevents Doxorubicin-Induced Cardiac Dysfunction in Rodent Model with Minimal Adverse Effects
Source: Pharmaceutics. 2022 Nov 28;14(12):2629. doi: 10.3390/pharmaceutics14122629 (PMC9788442; doi:10.3390/pharmaceutics14122629)
Supplement: Supplementary file 1 [file pharmaceutics-14-02629-s001.zip › pharmaceutics-2011379-supplementary.pdf]

Supplementary Material

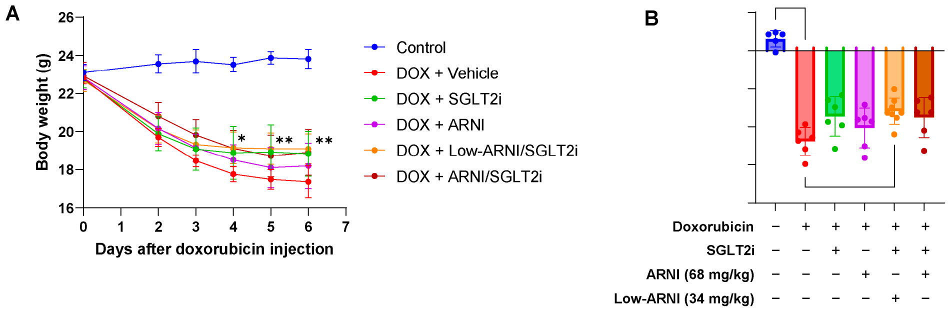

Supplemental Figure S1. Low-ARNI/SGLT2i treatment prevents body weight loss during acute doxorubicin administration**.** (A) Body weight changes over 6 days in the acute heart failure model (n=6–8/group). (B) Body weight percent changes from the baseline to day 6 after doxorubicin injection. * $p < 0.05$, ** $p < 0.01$.

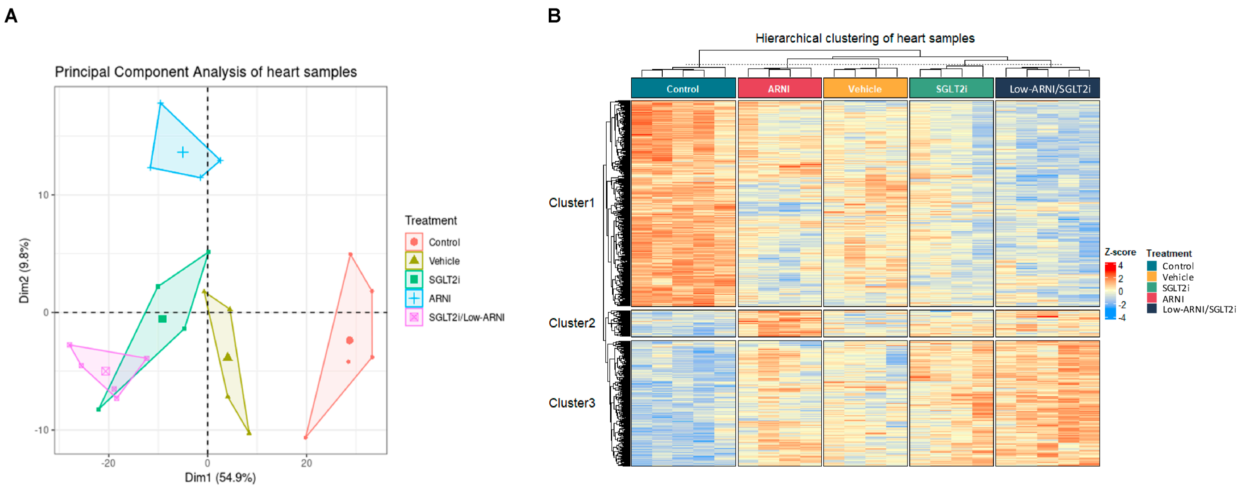

Supplemental Figure S2. Transcriptomic analysis during ARNI and SGLT2i treatment in the chronic doxorubicin model of heart failure. (A) Principal component scatter plot of transcriptomes of the heart samples from the control, doxorubicin+vehicle, doxorubicin+SGLT2i, doxorubicin+ARNI, and doxorubicin+low-dosage ARNI/SGLT2i groups (n=4–5 per group). (B) Hierarchical clustering and heatmap depicting the patterns of gene transcription in the control heart tissues, doxorubicin+vehicle, doxorubicin+SGLT2i, doxorubicin+ARNI, and doxorubicin+low-dosage ARNI/SGLT2i groups (n=4–5 per group).

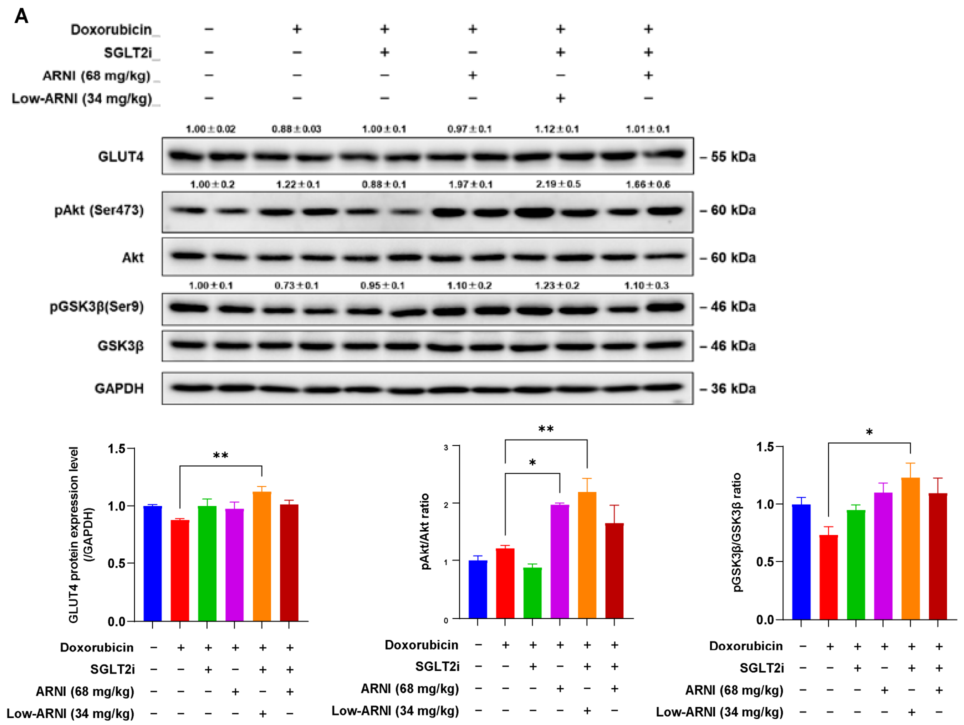

Supplemental Figure S3. ARNI and SGLT2i treatment effects on glucose metabolism re-partitioning in chronic doxorubicin-injected mice. (A) Representative immunoblots of GLUT4, pAkt, Akt, pGSK3β, and GSK3β and graphical quantification in the heart tissue at 9 weeks after doxorubicin injection in the indicated experimental group (n=4–5 per group). * $p < 0.05$, ** $p < 0.01$.

Supplemental Table S1. Primer sequences used in the present study

Genes	Forward	Reverse
36b4	TGCATCAGTACCCCATTCTATCA	AAGGTGTAATCCGTCTCCACAGA
Cpt1α	TCGAAACATCTACCATGCAGCA	CAGCATTCTTCGTGACGTTGG
Acadl	TCTTTTCCTCGGAGCATGACA	GACCTCTCTACTCACTTCTCCAG
Acsdvl	CTACTGTGCTTCAGGGACAAC	CAAAGGACTTCGATTCTGCCC
Acaa1a	TCTCCAGGACGTGAGGCTAAA	CGCTCAGAAATTGGGCGATG
Ehhadh	CAGCACTGGATGTGGATGAC	CATGACTGTGGCGATGGTAG
Hadha	TGCATTTGCCGCAGCTTTAC	GTTGGCCCAGATTTCGTTCA
Aldh3a2	CTTCCTGAATTGGCTTCTGC	AGCGGTTGCATGGTAAGAAC
Hmgcs2	ATACCACCAACGCCTGTTATGG	CAATGTCACCACAGACCACCAG
